# Medical Items

**Published:** 1908-04

**Authors:** 


### MEDICAL ITEMS

*Arteriosclerosis*: Autointoxication from faulty metabolism, and imperfect elimination ranks with gout and lead as a cause of high blood pressure. This, in time, leads to arteriosclerosis, cardiac hypertrophy and dilatation, mitral and aortic insufficiencies, cerebral apoplexy and retinal hemorrhage. Lowering the blood pressure is at once a preventive and curative measure. This is best accomplished by renal eliminations, and we know of no better remedy of this class than Alkalithia.

---

"Tongaline is a convenient and reliable remedy for that large class of painful complaints, whose etiology is so obscure as to present a veritable Chinese puzzle."

---

"In the treatment of all these diseases and diseased conditions resulting from the existence of the so-called rheumatic or uric acid diathesis, the action of salicylic acid from natural oil of wintergreen approaches so nearly to that of a specific as to be excelled only by that of cinchona on malarial toxaemia."

---

"The administration of the synthetic salicylic acid in full doses is almost always productive of unpleasant and often dangerous effects, such as irritation of the stomach, ringing of the ears and even dilirium; thus necessitating a diminution of the dose or a temporary suspension of treatment.

This is also the case with many extemporaneous prescriptions having salicylic acid as a base and combined with other indicated agents.

---

### THE WAY ITS DONE.

"Mother, may I get in the swim?"
"Yes, my darling daughter.
Buy your gowns from a Frenchy store,
And don't wear half you oughter."

*December Lippincott's*

### A NEW METHOD OF TESTING THE FUNCTIONS OF THE DIGESTIVE APPARATUS.

Einhorn (Therapeutic Gazette, January, 1908) submits a method for investigating the functions of the intestinal tract, the principle of which is the administration of test substances with the food and observation of the effects of the digestive fluids upon these substances.

Practically, this test is made as follows: Patients are given, in a gelatin capsule, a string of beads with the following substances attached thereto: catgut, fish-bone, meat, thymus, potato, mutton fat. After administering the capsule, every stool is examined with the stool-sieve until the bead-string has been recovered. If diarrhoea is present the sifting may not be necessary, as the bead-string can readily be seen (usually at the bottom of a glass vessel).

Under normal conditions the bead-string appears after one or two days. It is then rinsed in cold water and examined. If digestion is normal we find that catgut, meat, and potato (except the skin) disappear entirely, thymus and fat almost entirely, whereas the fish-bone usually disappears, but occasionally it may be present. The nuclei of the thymus always disappears. In pathological conditions deviations from the normal are observed, not only in regard to the time of recovery of the beads (disturbances of motility), but also in regard to the presence of the food substances (disturbances of the digestive function).

The author divides his cases of intestinal digestive disturbances into two groups:

1. Those of pure nervous intestinal dyspepsia. 2. Those of genuine intestinal dyspepsia.

In that great class of cases of intestinal dyspepsia, in which the starch digestion alone is disturbed, Taka-Diastase (Takamine) has proved of especial value.

---

Chemist (to poor woman)—You must take this medicine three times a day after meals.

Patient—But, sir, I seldom get meals these 'ard times.

Chemist (passing to next customer)—Then take it before.—*Glasgow Times.*

### MONGOLIAN BLUE PATCHES.

These occur on the skin in about 90 per cent. of Japanese children. They are dark blue in color, not raised above the surface, have an indefinite margin, and do not disappear on pressure. They are more marked in males than in females. Occasionally a child of European parents presents such patches, and the suggestion is that this means a previous crossing with some Mongolian strain.—*Dr. Ernest Jones.*

### BROMIDE INSURANCE.

Among the chemists who have testified to the purity of the salts entering the composition of Peacock's Bromides, particularly as to its extraordinary freedom from chlorides and the absence of other usual impurities, are names of such eminent men as Edward H. Keiser, Ph. D., Professor of Chemistry Washington University; H. Helblng, F. C. S., and F. W. Passmore, Ph. D., of London, England; Charles E. Caspari, Ph. D., Professor Chemistry St. Louis College of Pharmacy, and Edward Gudeman, Ph. D., Chicago, Ill.

Thus when a physician prescribes Peacock's Bromides he has the benefit of Bromide Insurance, as the preparation can be depended on to give the best possible results obtainable from bromides.

Peacock's Bromides is a mixture of bromides of Potassium, Sodium, Ammonium, Calcium and Lithium, 15 grains combined in each fluid drachm and equivalent in dosage to 15 grains of potassium bromide.

### GRIM WARNING TO GIRLS.

We recently read a horrible story of a young lady who thoughtlessly jerked her head back suddenly to keep from being kissed, and broke her neck. This should be a terrible warning to girls not to jerk back. In fact, it would be better to lean forward just a little.—*Caldwell, (Okla.) Advance.*

